# Recruiting and retaining community-based participants in a COVID-19 longitudinal cohort and social networks study: lessons from Victoria, Australia

**DOI:** 10.1186/s12874-023-01874-z

**Published:** 2023-02-27

**Authors:** Thi Nguyen, Alexander J. Thomas, Phoebe Kerr, Ashleigh C. Stewart, Anna Lee Wilkinson, Long Nguyen, Aimée Altermatt, Kathryn Young, Katherine Heath, Anna Bowring, Stephanie Fletcher-Lartey, Dean Lusher, Sophie Hill, Alisa Pedrana, Mark Stoové, Katherine Gibney, Margaret Hellard

**Affiliations:** 1 Disease Elimination, Burnet Institute, 85 Commercial Road, Melbourne, VIC 3004 Australia; 2 School of Public Health and Preventive Medicine, Monash University, Melbourne, VIC 3004 Australia; 3 Swinburne University of Technology, Hawthorn, VIC 3122 Australia; 4 La Trobe University, Bundoora, VIC 3086 Australia; 5 Department of Infectious Diseases, University of Melbourne, at the Peter Doherty Institute for Infection and Immunity, Parkville, VIC 3052 Australia; 6 Department of Infectious Diseases, The Alfred and Monash University, Melbourne, VIC 3004 Australia

**Keywords:** Covid-19, Longitudinal cohort study, Attrition bias

## Abstract

**Background:**

Longitudinal studies are critical to informing evolving responses to COVID-19 but can be hampered by attrition bias, which undermines their reliability for guiding policy and practice. We describe recruitment and retention in the Optimise Study, a longitudinal cohort and social networks study that aimed to inform public health and policy responses to COVID-19.

**Methods:**

Optimise recruited adults residing in Victoria, Australia September 01 2020–September 30 2021. High-frequency follow-up data collection included nominating social networks for study participation and completing a follow-up survey and four follow-up diaries each month, plus additional surveys if they tested positive for COVID-19 or were a close contact. This study compared number recruited to a-priori targets as of September 302,021, retention as of December 31 2021, comparing participants retained and not retained, and follow-up survey and diary completion October 2020–December 2021. Retained participants completed a follow-up survey or diary in each of the final three-months of their follow-up time. Attrition was defined by the number of participants not retained, divided by the number who completed a baseline survey by September 302,021. Survey completion was calculated as the proportion of follow-up surveys or diaries sent to participants that were completed between October 2020–December 2021.

**Results:**

At September 302,021, 663 participants were recruited and at December 312,021, 563 were retained giving an overall attrition of 15% (*n* = 100/663). Among the 563 retained, survey completion was 90% (*n* = 19,354/21,524) for follow-up diaries and 89% (*n* = 4936/5560) for monthly follow-up surveys. Compared to participants not retained, those retained were older (t-test, *p* <  0.001), and more likely to be female (χ^2^*, p* = 0.001), and tertiary educated (χ^2^*, p =* 0.018).

**Conclusion:**

High levels of study retention and survey completion demonstrate a willingness to participate in a complex, longitudinal cohort study with high participant burden during a global pandemic. We believe comprehensive follow-up strategies, frequent dissemination of study findings to participants, and unique data collection systems have contributed to high levels of study retention.

**Supplementary Information:**

The online version contains supplementary material available at 10.1186/s12874-023-01874-z.

## Introduction

On March 11 2020, Coronavirus Disease 2019 (COVID-19) was declared a pandemic by the World Health Organization [1]. In Australia, the first phases of the COVID-19 pandemic response in 2020 and 2021, prior to achieving high vaccine coverage, focussed on preventing transmission through restricting movement in the community, and influencing individual behaviour [2]. As well there were limits on international arrivals and departures, and travel between Australian jurisdictions [3]. Victoria, Australia’s second most populous state, implemented a number of such measures (e.g., school closures, stay-at-home directives, and density limits on indoor and outdoor public spaces). Preventive policy evolved throughout 2020 as improved understanding of the route of COVID-19 transmission emerged [4, 5], with vaccines then becoming available in Australia in February 2021 and widespread coverage (i.e., 80% of Australians aged 16 years or over had received two COVID-19 vaccines) achieved by November 2021 [6]. This ongoing and adaptive public health response underscores the importance of longitudinal studies’ ability to measure changes in behaviour and public sentiment in response to changing measures over time. These data can be used to inform public health policy and implementation strategies by assessing feasibility, acceptability and community support for interventions [7–9].

The strength of a longitudinal study to measure change at an individual level can be limited by lost-to-follow-up (i.e., attrition). Substantial or “biased” lost-to-follow-up can compromise the validity, reliability, and generalisability of study findings [9]. Longitudinal studies can involve intensive data collection and extended follow-up periods, increasing the risk of participants becoming disengaged or uncontactable due to changes in personal circumstances or personal details. These factors, alongside risk of illness or deaths, can result in missing data that is either once-off, across a short period, or permanent [10, 11]. However, there is limited evidence on participation in COVID-19-related longitudinal studies. The Optimise Study (Optimise) is a longitudinal cohort and social networks study conducted in Victoria, Australia. Optimise is characterised by the recruitment of “seeds” and their social networks, alongside high frequency and detailed data collection that included collection of data on participants’ contact with their social network. Optimise placed a high data collection burden on participants; data collection was frequent, participants were asked to provide sensitive data (names and contact details) of their social networks, have their social networks approached to participate in a study alongside them, and describe in detail their contact with people. Assessing participant recruitment and retention in a study with these design features and can help to inform future social research in the context of a fast-moving pandemic. This study uses data from Optimise to: 1) describe recruitment and survey completion retention, and 2) test for differences in baseline characteristics between those retained in the study and not retained.

## Methods

### Study design

Optimise is a longitudinal cohort and social network study that aimed to recruit approximately 1000 community-based Victorians. Recruitment commenced on September 012020 then ceased September 302,021. Optimise initially aimed to recruit from two groups; Group one were people infected with COVID-19 (recruited soon after infection) or people notified as “close contacts” of a person with COVID-19 and Group two were people not currently infected with COVID-19 but at higher risk of infection and/or adverse outcomes from COVID-19 infection and/or public health measures, for example negative changes to employment conditions, housing, or access to primary healthcare. However, low case numbers in Victoria in 2020 and much of 2021 [12] meant that recruitment into Group one was limited. Henceforth, this study describes recruitment and retention of the Group two participants only.

Optimise was designed to use social network analysis to inform the response to COVID-19 and therefore used a snowball sampling methodology for recruitment. An initial set of seeds was purposively sampled from selective groups (Layer 0) with seeds recruited after completing an ‘expression of interest’ in the study form, advertised through paid and unpaid social media advertisements and flyers promoted through community and industry groups, community-based organisations, and social and professional networks. At recruitment, seeds were asked to nominate “Key People”, with the aim of recruiting the Key People into the study. Key People were described as people in the participant’s life with whom they would discuss personal matters, receive practical support, co-workers with whom they frequently interact, and/or people with whom they share activities (i.e., hobbies, sport, and/or relatives). Up to eight Key People per participant were contacted by research staff, using contact details provided by seeds, and research staff would disclose the name of the seed that had nominated them, and invite them to participate in the study. At study commencement, there were five research staff engaged specifically implement study recruitment and collect data. Two “layers” of Key People were recruited; that is, a seed (Layer 0) would nominate Key People (Layer 1), who if recruited, would then nominate Key People (Layer 2).

### Participants

Participants were eligible for recruitment if they were 18 years or older, resided in Victoria, were willing and able to provide informed consent, were able to provide a valid email and phone number, and had access to the internet to complete online surveys or be available for phone interviews.

Seed (Layer 0) target numbers were initially set and then revised as the study progressed to respond to the dynamic nature of the pandemic and emerging priority populations, and to respond to fewer Key People (Layers 1 and 2) being recruited than planned. The final seed target was 210: 20 healthcare workers, 20 aged-care workers, 20 people working in high-risk workplace (factories/warehouses), 30 people from regional town centres, 40 people with pre-existing chronic conditions, 40 people speaking a language other than English at home, 20 young people (aged 18–24 years), and 20 people working as part of the program to quarantine people arriving from overseas.

### Data collection

**Fig. 1:**
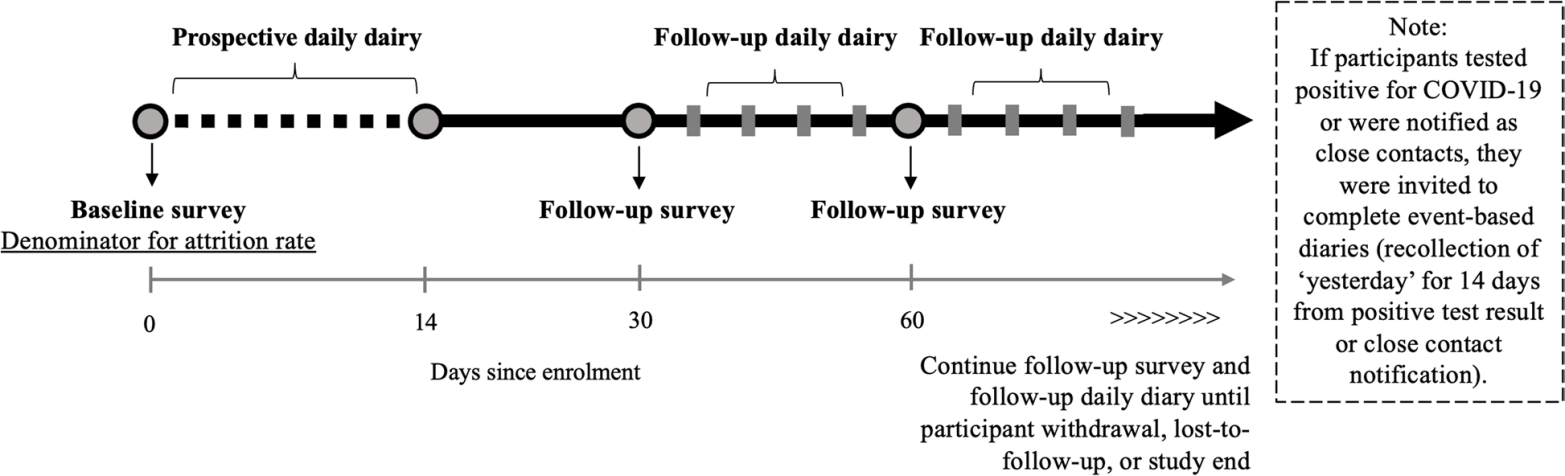
Typical study timeline for participants in the Optimise Study

Optimise collected data across a broad range of domains, including socio-demographics, work and study circumstances, health behaviours, access to services and information, social connectedness, mental health, knowledge of COVID-19, attitudes towards COVID-19 prevention measures, and contact with people. Optimise aimed to provide comprehensive and timely data to inform the public health response to COVID-19, including providing data to inform social networks and mathematical modelling [13]. Therefore, Optimise deployed intensive and high-frequency data collection using a suite of tools including once-off surveys, repeated follow-up surveys, repeated follow-up diaries, and event-based diaries. Automated reminder emails or SMS were sent to participants with pending surveys due for completion each Monday at 8 AM (see Supplementary Fig. 3). Over the study, participants completed 16 data collection tools at baseline (one Key People nomination, one baseline survey, then 14 prospective daily diaries) then five data collection points each month (one monthly follow-up survey and four follow-up diaries) with optional additional event-based diaries (Fig. 1).

#### Recruitment and baseline—once-off surveys

At study recruitment, all participants completed a recruitment interview including study consent and nomination of Key People via a phone interview with a researcher. Participants could then opt to complete subsequent surveys either online (self-complete) or via phone interview (interview administered) or a mixture of both (partial self-complete). After the nomination of Key People, participants completed the baseline survey and commenced the baseline prospective daily diary (recollection of “yesterday”) and completed a diary each day from day 1 to 14 after the day of recruitment.

#### Follow-up—repeated survey and diaries

Follow up commenced 28 days after recruitment. Participants were invited via email, SMS, or by a phone call from research staff every 28 days (henceforth referred to as monthly follow-up) to complete a follow-up survey (recall period of past month). Timing of survey invitations aligned with individuals’ recruitment date, as opposed to surveys being sent to all participants on the same date each month. Participants were also invited to complete four follow-up diaries in a month (recollection of “yesterday”, scheduled randomly for two weekdays and two weekend days) to capture timely data on experiences of COVID-19 testing, COVID-19 related symptoms, being a “close contact” (i.e., notified by health department they were a close contact of a person who tested positive for COVID-19), and detailed information on contact with people between monthly follow-up surveys.

#### Event-based diaries

To capture data on participants’ experiences of quarantine and isolation and adherence to public health measures, if participants reported in a monthly follow-up survey or follow-up diary that they had tested positive for COVID-19 or were identified as a close contact, they were invited to repeat the prospective daily diary (recollection of “yesterday” for 14 days) they completed at baseline, starting on the day of the positive test result or notification of being a close contact.

### Reimbursements

Participants were reimbursed $35 (AUD) for the baseline survey and $15 if at least 10/14 baseline prospective daily diaries were completed. Then each month of follow-up, participants received $2.50 for each follow-up daily diary completed and $25 for each follow-up survey completed. If participants who tested positive for COVID-19 or were notified they were a close contact during follow-up elected to complete an “event-based” prospective daily diary each day for 14 days as an additional task, they were reimbursed $20.

This was simplified in December 2020 to $50 for all recruitment and baseline data collection, $35 each month for completion if any follow-up was completed in that month (minimum one follow-up diary), and $20 for any additional prospective daily diary (14 days) if participants tested positive for COVID-19 or were close contacts. In total, if a participant was retained for 12 months, and completed a baseline and at least one follow-up survey a month, they would be reimbursed $470. All reimbursements were by electronic gift vouchers.

### Statistical analysis

#### Recruitment and baseline surveys completion

We reported monthly recruitment intake between recruitment commencement on September 012020 and recruitment end on September 302,021 against the final seed target numbers and assessed internal selection bias by summarising cohort baseline characteristics.

To indicate participant burden at recruitment, we described survey completion times (minutes between a survey being opened and successfully submitted) for baseline surveys (Key People nomination, baseline survey, baseline prospective daily diary) for all participants recruited between September 012020 and September 302,021. We reported, using summary statistics, the mean, standard deviation (SD), median, and interquartile range (IQR) of completion time, for each type of survey.

#### Attrition and cohort characteristics

We described Optimise’s attrition rate for all participants recruited. Because Optimise had rolling recruitment up to September 302,021, we allowed participants at least 3 months of follow-up time but a maximum of 12 months’ follow-up time. We assessed the rate of attrition in the cohort by classifying participants as retained or not retained (lost-to-follow-up or withdrawn). All participants retained completed a follow-up diary or follow-up survey in each of the last 3 months of their follow-up. Those who did not meet the criteria for retained were considered lost-to-follow-up (“not retained”); in addition, participants who withdrew from the study at any point by contacting the study team (either by phone or email) were also defined as “not retained”. The overall attrition rate was calculated as the number of participants not retained divided by the number of participants who completed a baseline survey from September 012020–September 302,021. Differences in baseline characteristics between retained and not retained participants were assessed using *t*-test for continuous variables (because they were normally distributed) and chi-squared test for categorical variables, with results reported as a *p*-value.

#### Follow-up surveys completion

To assess follow-up completion rates (follow-up diaries and monthly surveys) among retained participants, we defined a completed survey as all questions answered and the survey successfully submitted to the data capture system (NetCollect [14]). Partially completed surveys were therefore considered incomplete. For the denominator of completion, participants received an invitation for each survey from the data capture system (NetCollect [14]) and every invitation was recorded and available for analysis. We defined completion rates as the number of completed surveys divided by the total number of invitations. We reported completion rates as proportions, for retained participants for both follow-up diaries and follow-up surveys. Completion rates of follow-up surveys and diaries were estimated for each month starting October 01 2021 (1 month after recruitment commenced, September 012020) until this study’s observation period end (December 312,021). To assess the relationship between monthly completion rates and the time a participant had been in the study, a linear regression model was fit using completion rate and time (months) since recruitment. We reported the expected (mean) percentage (95% confidence intervals) change in completion rate per month since recruitment for each type of follow-up tool (survey or diary).

To assess completion times, we also reported the mean completion time, standard deviation (SD), median, and interquartile range (IQR) for each type of follow-up survey.

All analyses were conducted using R 4.1.0 [15] and RStudio 1.4.1 [16] under macOS Monterey 12.2.1.

## Results

### Recruitment and baseline surveys completion

A total of 1166 people completed the expression of interest of whom 663 people were recruited into the Optimise study between September 012020 and September 302,021. Within approximately 10 months from commencing recruitment, most of the seed population targets were reached.

**Fig. 2:**
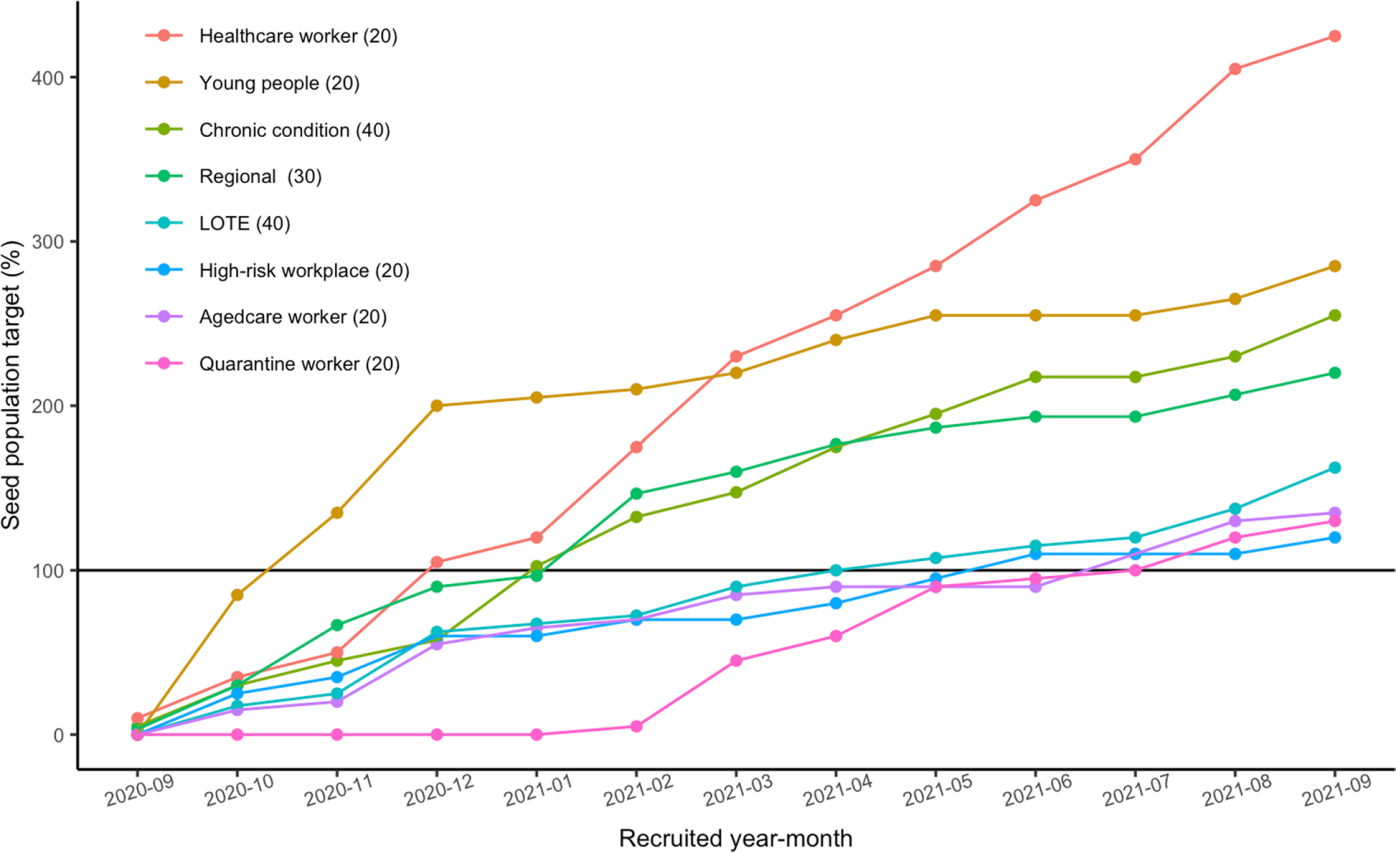
Monthly recruitment intake against the seed population targets, September 012020–September 302,021

Seed targets for young people were reached within approximately 1 month, and for healthcare workers, people with chronic conditions, and people living in regional Victoria reached within 4 months (Fig. 2). Remaining seed targets for people speaking a language other than English at home (LOTE), people working in high-risk workplaces, aged-care workers, and people working in quarantine facilities were reached between 8 and 11 months from commencing recruitment. For some groups, seed targets were exceeded where study investigators deemed it appropriate (Fig. 2).

Among the 663 recruited participants in this study, the time to completion of the baseline surveys showed each Key People nomination (*n* = 658) taking a mean duration of 18 minutes (SD = 11.4, median = 16, IQR 12.0–22.0) to complete, baseline survey (*n* = 663) a mean of 36 minutes (SD = 26.6, median = 30.0, IQR 23.0–41.0) to complete, and each baseline prospective daily diary (*n* = 8361) taking a mean of 5 minutes (SD = 12.7, median = 3.0, IQR 2.0–6.0) to complete.

### Attrition rate and cohort characteristics

Of the 663 participants who were recruited into the Optimise study, by December 312,021, 17 participants had withdrawn and 83 participants were lost-to-follow-up, giving an overall attrition rate of 15% (*n* = 100/663). Participant flow chart and number of people recruited each month is available in Supplementary (Supplementary Figs. 1 and 2 respectively).

Among 563 retained participants, the mean age at baseline was 46 years (SD = 17.2), 75% identified as female (420/563), 28% were born outside of Australia (*n* = 159/563), 20% were living in regional Victoria (113/563), and 83% had completed tertiary education (*n* = 467/563; Table 1).

**Table 1:** Baseline characteristics among retained and not retained participants, Optimise, September 012020 to December 312,021, (*N* = 663)

	Retained (***n*** = 563)	Not retained (***n*** = 100)	***p***-value
Age, mean years (SD)	45.5 (17.2)	39.1 (16.5)	< 0.001^*^
Female (vs. non-female)	420 (74.6%)	59 (59.0%)	**0.001**^**†**^
Born outside Australia (vs. Born in Australia)	159 (28.2%)	29 (29.0%)	0.877^**†**^
Language other than English (vs. English)	46 (8.2%)	12 (12.0%)	0.212^**†**^
Non-permanent resident (vs. permanent resident)	46 (8.2%)	10 (10.0%)	0.544^**†**^
Residing in regional Victoria (vs. metropolitan)	113 (20.1%)	23 (23.0%)	0.504^**†**^
Tertiary education (vs. non-tertiary educated)	467 (82.9%)	73 (73.0%)	**0.018**^**†**^
Employment status			**0.043**^§^
Full-time	190 (33.7%)	27 (27.0%)	
Part-time	106 (18.8%)	17 (17.0%)	
Casual	87 (15.5%)	22 (22.0%)	
Self-employed	23 (4.1%)	8 (8.0%)	
Currently looking for job	27 (4.8%)	7 (7.0%)	
Not currently looking for job	18 (3.2%)	5 (5.0%)	
Retired	86 (15.3%)	10 (10.0%)	
Carer	4 (0.7%)	3 (3.0%)	
Other	22 (3.9%)	1 (1.0%)	
Seed population (vs. Key People)	303 (53.8%)	54 (54.0%)	0.973^**†**^
Survey completion method^a^			
Self-complete	543 (96.4%)	92 (92.0%)	
Partial self-complete	2 (0.4%)	1 (1.0%)	
Phone administrated	18 (3.2%)	7 (7.0%)	
Source of recruitment^a^			
Social media	217 (38.5%)	35 (35.0%)	
Work	20 (3.6%)	5 (5.0%)	
Friends	312 (55.4%)	57 (57.0%)	
Health service	3 (0.5%)	0 (0.0%)	
Other	11 (2.0%)	3 (3.0%)	

*SD* Standard deviation; *p*-value computed using ^*^t-test (without correction) for continuous, normally distributed, variables and ^†^chi-squared test for categorical variables. ^a^Test for difference omitted due to small cell sizes. ^§^Fisher’s exact test for difference due to small cell sizes

Retained participants were on average older than participants who were lost-to-follow-up, with a mean age difference of 6.4 years (*p* <  0.001; Table 1). Females (*p* = 0.001), and tertiary educated participants (*p* = 0.018) were more likely to be retained in the study (Table 1).

### Follow-up survey completion

Among the 563 retained participants, the mean time to complete monthly follow-up surveys (*n* = 4936) was 22 minutes (SD = 21.8, median = 18.0, IQR 14.0–25.0). Participants spent on average 3 minutes (SD = 4.7, median = 2.0, IQR 1.0–4.0) to complete follow-up diaries (*n* = 19,354).

On average, retained participants (*n* = 563) completed 90% (19,354/21,524) of invited follow-up diaries (average 4 invitations per calendar month), and 89% (4936/5560) of invited monthly follow-up surveys.

**Fig. 3:**
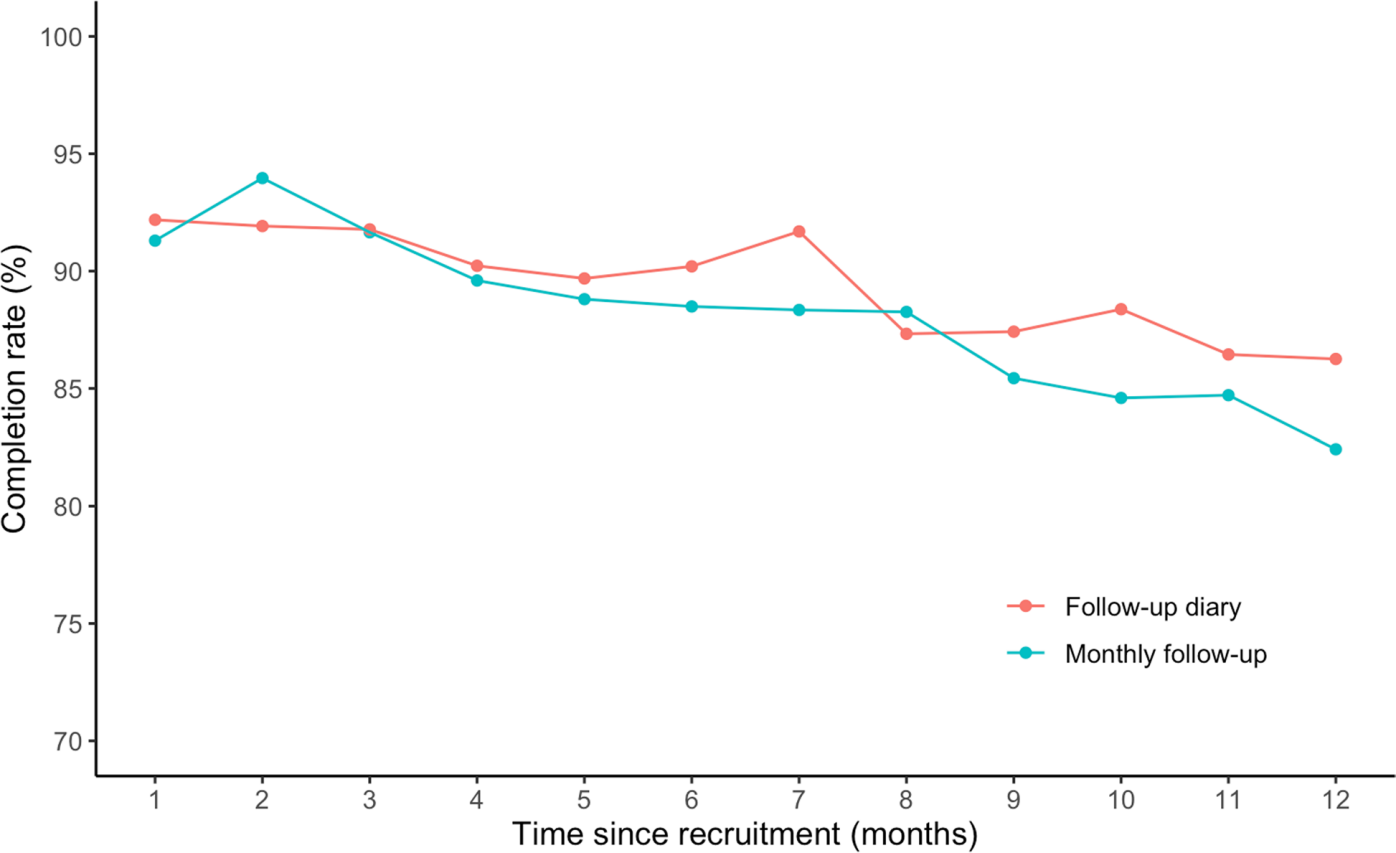
Completion rates of active participants over time since recruitment (months), October 012020–December 312,021. In this study participants could contribute a minimum of three-months and a maximum of 12-months follow-up time

Figure 3 displays follow-up diary and follow-up survey completion rates among retained participants over time since recruitment. After 1 month of follow-up, retained participants completed 92% (2076/2252) of invited follow-up daily diaries and 91% (514/563) of monthly follow-up surveys. Among participants that completed 12 months of follow-up (*n* = 211), completion rates declined to 86% (797/924) for follow-up diaries and 82% (211/256) for monthly follow-up surveys at the 12-month point. On average, the completion rate declined 0.6% per month since recruitment (95% CI -0.7, − 0.3%) for diaries and declined 0.9% per month since recruitment (95% CI -1.1, − 0.7%) for surveys.

**Fig. 4:**
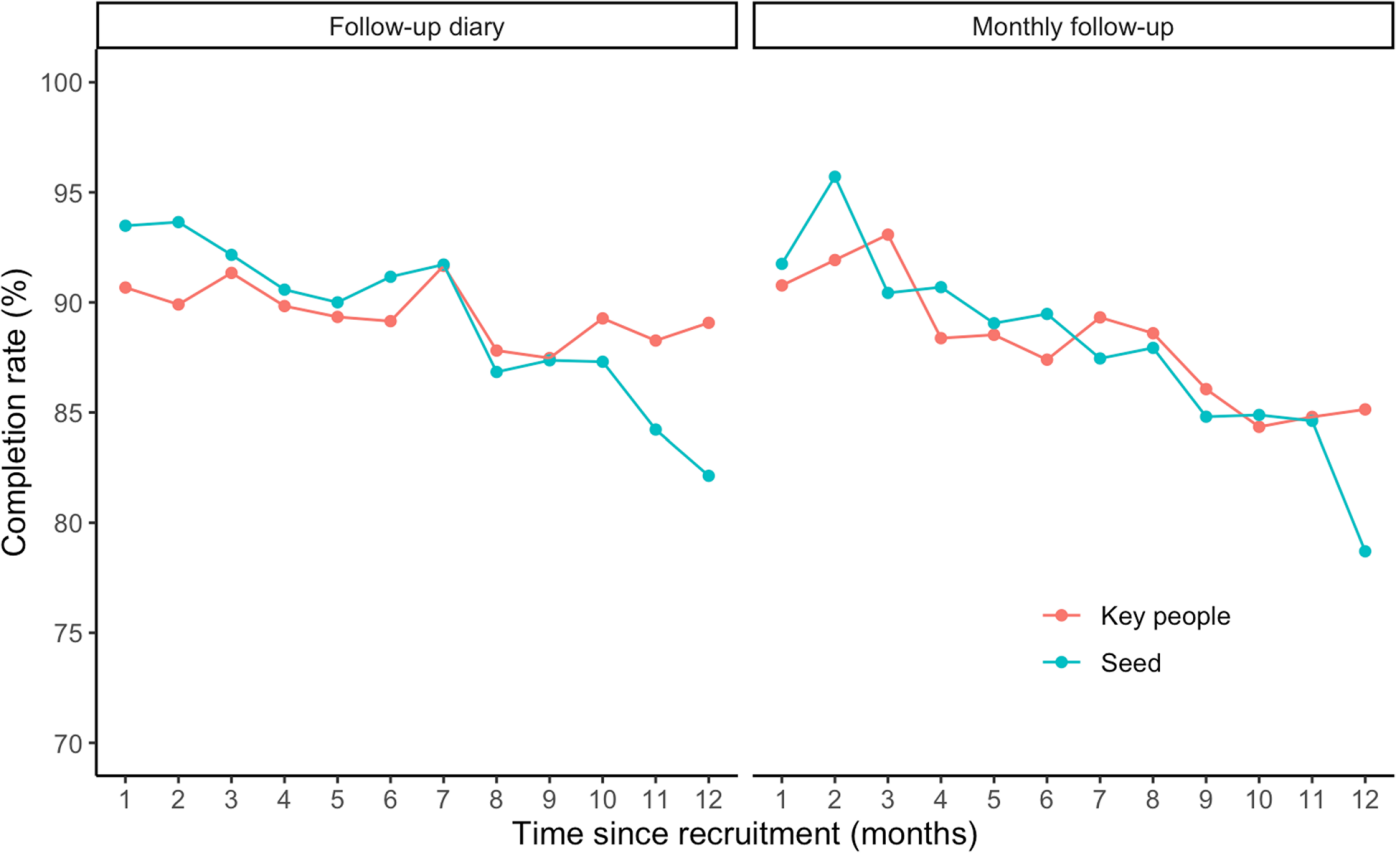
Completion rates of participants over time since recruitment (months), stratified by Key people and seed participants, October 01 2020–December 312,021. In this study participants could contribute a minimum of three-months and a maximum of 12-months follow-up time

Figure 4 displays the monthly completion rates of seed participants (i.e., a participant recruited to the study without any referral or nomination by other participants) and Key People (i.e., people nominated by a study participant and recruited to the study) for each survey type. For follow-up diaries, the completion rate declined, on average, 0.9% per month since recruitment (95% CI: − 1.2, − 0.7%) for seed participants and declined 0.2% per month since recruitment (95% CI: − 0.4, 0.0%) for Key People participants. For monthly follow-up surveys, there were no significant differences (*p*-value = 0.3) in completion rates of seed participants and Key People except for the month 12 since recruitment where the difference was 6.4%.

## Discussion

This study demonstrates successful recruitment of participants and a high level of retention in a complex cohort and social networks study during the COVID-19 global pandemic. Minimal attrition bias in the Optimise study supports the validity of previously published study findings, which have provided important, publicly available evidence for community and government responses to COVID-19 [17]. The high study retention and willingness to participate in Optimise may, in part, be due to a peak in community interest in public health research as a result of the pandemic and the desire to contribute to the response and exert some control during a time of uncertainty [18]. Further, study reimbursements, collection of detailed contact tracing information and comprehensive approaches to follow-up, including automated reminders for survey completion and the involvement of experienced data collectors, likely contributed to the high study retention rate.

The low attrition (15%) and high completion rates (~ 90%) over the 12-month observation period were in spite of the participant burden of the study (repeated and high frequency surveys) [19, 20]. Based on literature of longitudinal cohort studies, which would typically have annual follow-up, we reasonably expected attrition to be closer to 30% [19]. Of note though, a previous population cohort study during the COVID-19 pandemic reported a similar study attrition with lower frequency data collection (one follow-up survey), with a study from Munich reporting 17% attrition between April 2020 and January 2021 [21]. Some explanations for our results may point to a selection bias in Optimise; participants were mostly tertiary educated, employed, English was their primary language spoken at home, and they had access to technology. Participants also had the personal resources (e.g., time, technology) to participate. Similarly, participants retained were older on average, more likely to be women, and more likely to be tertiary educated. Our participants may have had a pre-existing appreciation for research and/or were “community-minded”, driving their interest in participating in COVID-19 research. Further, Optimise commenced when there were high levels of uncertainty about the pandemic (that is, vaccines and other therapeutics were not available), therefore, participants were likely aware of the urgent need for public health research, the value of their participation, and the importance of evidence because they were at higher risk of COVID-19 infection and/or poor outcomes. Also, when considering participation in health research, it is important to consider the broader social context including that Australia has a universal health care system, the government provided additional financial support to people during the pandemic, and generally, there was support for public health measures to control COVID-19, indicated by adherence with public health measures. Whilst low attrition mitigates study bias and increases confidence in findings, some bias towards “aware and able” participants does need to be considered in future uses of the data.

A range of comprehensive follow-up strategies were implemented over the course of the Optimise study. Maintaining contact with participants, through either or both online or research staff data collection, monthly reimbursement, and/or directly disseminating reports of findings, likely contributed to the low attrition rate observed. Such strategies included a team of experienced data collectors who, in most cases, followed the same study participants from enrolment and maintained ongoing contact throughout the duration of the study, providing a personalised approach to participant follow-up and/or responding to participant enquiries. The high level of functionality available via the data capture system (NetCollect) also likely contributed to the low attrition rate. Notably, automatic reminders and survey links were sent to participants’ nominated email address or phone number or both, which is an approach previously demonstrated to increase study retention [22]. Further, participant “dashboards” (see Supplementary Figs. 4 and 5) were available to research staff, which displayed the number of completed surveys and participant contact information, allowing ease of participant follow-up when surveys were marked as incomplete. When non-completion was noted, participants were followed up via phone and offered support to complete surveys, including interviewer-administered survey completion. The breadth of follow-up strategies and flexibility of data collection methods (via phone or self-completion) has likely contributed to the low attrition in the Optimise study, with such methods previously highlighted as crucial in maximising cohort study retention [19]. Finally, Optimise distributed a monthly newsletter and provided access to a study website noting study updates, recruitment numbers, and study findings via reports and news articles, which was intended to inform participants of study progress and keep participants engaged in the research.

This study has some limitations to consider when interpreting the findings. The social network component of the study design meant seed participants were from priority groups, therefore, the Optimise sample was not intended to be representative of the general population of Victoria. Oversampling of key groups increased statistical power to detect differences in the outcomes between these groups therefore was a strength of the study however limits generalisability. It is important to note that Optimise disproportionately recruited women, further limiting the generalisability of the findings. Further, the study was limited to adults therefore it was outside the scope of this study to understand recruitment and retention of children in longitudinal studies. We adopted a broad definition of study retention (completion of any follow-up diary out of four possible diaries invited to in a month or a follow-up survey) and a stricter definition may result in lower retention rates. Further, some participants recruited later in study had only three-months of follow-up and therefore our estimate of retention may be an over-estimate as given more follow-up time, they may withdrawn or been lost-to-follow-up. Though in the context of Optimise, which has a high burden on participants, and considering our definition meant participants needed to have completed consecutive surveys in the final 3 months of follow-up, our definition is still a reasonable assessment of the success of data collection and prevention of missing data. This study presents a descriptive analysis reporting on correlations between study attrition and participant characteristics and therefore cannot assess causal relationships. While this study explores some differences in participants retained and lost-to-follow-up, it possible that there were unmeasured confounders that may have affected participants’ follow-up status. Further, reason/s for withdrawal was not systematically collected, therefore whilst we compared baseline characteristics of those retained and not retained, an in-depth analysis of those withdrawn was outside the scope of this study. Lastly, the impact of social influence on participants’ social networks was not assessed, which may have affected individuals’ continued participation in a complex longitudinal social network study. Social network analysis findings suggest that including social network methodology in a study may strongly influence study retention, especially for longitudinal cohorts; it was beyond the scope of this study to examine this in detail, but future research by the investigators will examine this [23].

## Conclusion

Our study demonstrates, during a global pandemic, there was a high willingness to engage in a longitudinal study for COVID-19. While retention was associated with age, gender, location, and education, comprehensive follow-up strategies, transparency with study findings and the unique data collection system likely contributed to low overall study attrition. The data collected and findings drawn from the study have been used to directly inform the Victorian government response on COVID-19 policy and practice.

## Supplementary Information

**Additional file 1: Supplementary Figure 1.** Optimise study flow diagram.

**Additional file 2: Supplementary Figure 2.** Number of new participants recruited into the study by month and by seed and Key People participants, Optimise Study, Victoria, Australia, September 01, 2020–September 30, 2021.

**Additional file 3: Supplementary Figure 3.** An example of survey reminder email sent to a Optimise participant.

**Additional file 4: Supplementary Figure 4.** An example of participant “dashboard” of a Optimise participant.

**Additional file 5: Supplementary Figure 5.** An example of survey calendar shows survey completion of a Optimise participant.
