# Supplementary material for: Recruiting and retaining community-based participants in a COVID-19 longitudinal cohort and social networks study: lessons from Victoria, Australia
Source: BMC Med Res Methodol. 2023 Feb 27;23:54. doi: 10.1186/s12874-023-01874-z (PMC9969937; doi:10.1186/s12874-023-01874-z)
Supplement: Supplementary file 1 — Additional file 1: Supplementary Figure 1. Optimise study flow diagram. [file 12874_2023_1874_MOESM1_ESM.docx]

|  |  | Total number of participants consented into Optimise  n=716 |  |  |
| --- | --- | --- | --- | --- |
|  |  |  |  |  |
|  |  | Total number of participants who completed Key People  n=694 |  |  |
|  |  |  |  |  |
| Participants who completed retrospective daily dairy (for Group one participants [COVID infected] only, recollection of each day of the past 14 days at once, day -14 to 0)  n=7 |  | Total Number of participants who completed baseline survey (day 0)  n=663  Denominator for attrition rate. |  | Participants who completed prospective at least one daily dairy (for Group one participants (recollection of ‘yesterday’ for 14 days, day 1 to 14)  n=650 |
|  |  |  |  |  |
|  |  | Total number of participants who completed a follow-up survey (recollection of past month)  n=662 |  |  |
|  |  |  |  |  |
| Note  Participants retained in the study continue completing the follow-up survey (recollection of past month) and the follow-up diary (recollection of ‘yesterday’, a randomised day in the past 7 days) until study withdrawal, lost-to-follow-up, or study end. |  | Total number of participants who completed a follow-up diary (recollection of ‘yesterday’, a randomised day in the past 7 days)  n=662 |  | If participants tested positive for COVID-19 or were notified as close contacts, they were invited to repeat the prospective daily diary (recollection of ‘yesterday’ for 14 days from positive test result or close contact notification).  Total number of people who completed a prospective daily diary during follow-up  n=2 |

**Supplementary Figure 1.** Optimise study flow diagram. Light grey shading represents study components completed once and conducted via a phone interview with research staff. Median grey shading with solid boarder represents once off surveys and diaries completed via phone interview with research staff or self-completed via online survey. Dark grey shading represents follow-up surveys and diaries completed via phone with research staff or self-completed via online survey. Medium grey shading with broken line boarder represents option to completed prospective daily diary if tested positive for COVID-19 or notified as a close contact.
