# Supplementary material for: Recruiting and retaining community-based participants in a COVID-19 longitudinal cohort and social networks study: lessons from Victoria, Australia
Source: BMC Med Res Methodol. 2023 Feb 27;23:54. doi: 10.1186/s12874-023-01874-z (PMC9969937; doi:10.1186/s12874-023-01874-z)
Supplement: Supplementary file 2 — Additional file 2: Supplementary Figure 2. Number of new participants recruited into the study by month and by seed and Key People participants, Optimise Study, Victoria, Australia, September 01, 2020–September 30, 2021. [file 12874_2023_1874_MOESM2_ESM.docx]

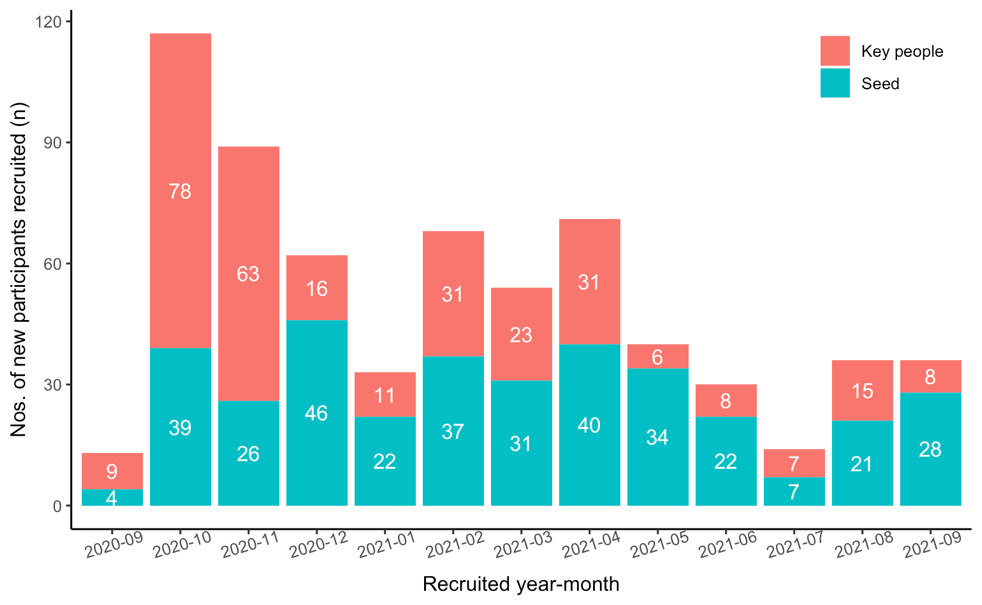

**Supplementary Figure 2.** Number of new participants recruited into the study by month and by seed and Key People participants, Optimise Study, Victoria, Australia, September 01, 2020–September 30, 2021.
