# Supplementary figures and images for: Recruiting and retaining community-based participants in a COVID-19 longitudinal cohort and social networks study: lessons from Victoria, Australia
Source: BMC Med Res Methodol. 2023 Feb 27;23:54. doi: 10.1186/s12874-023-01874-z (PMC9969937; doi:10.1186/s12874-023-01874-z)

*
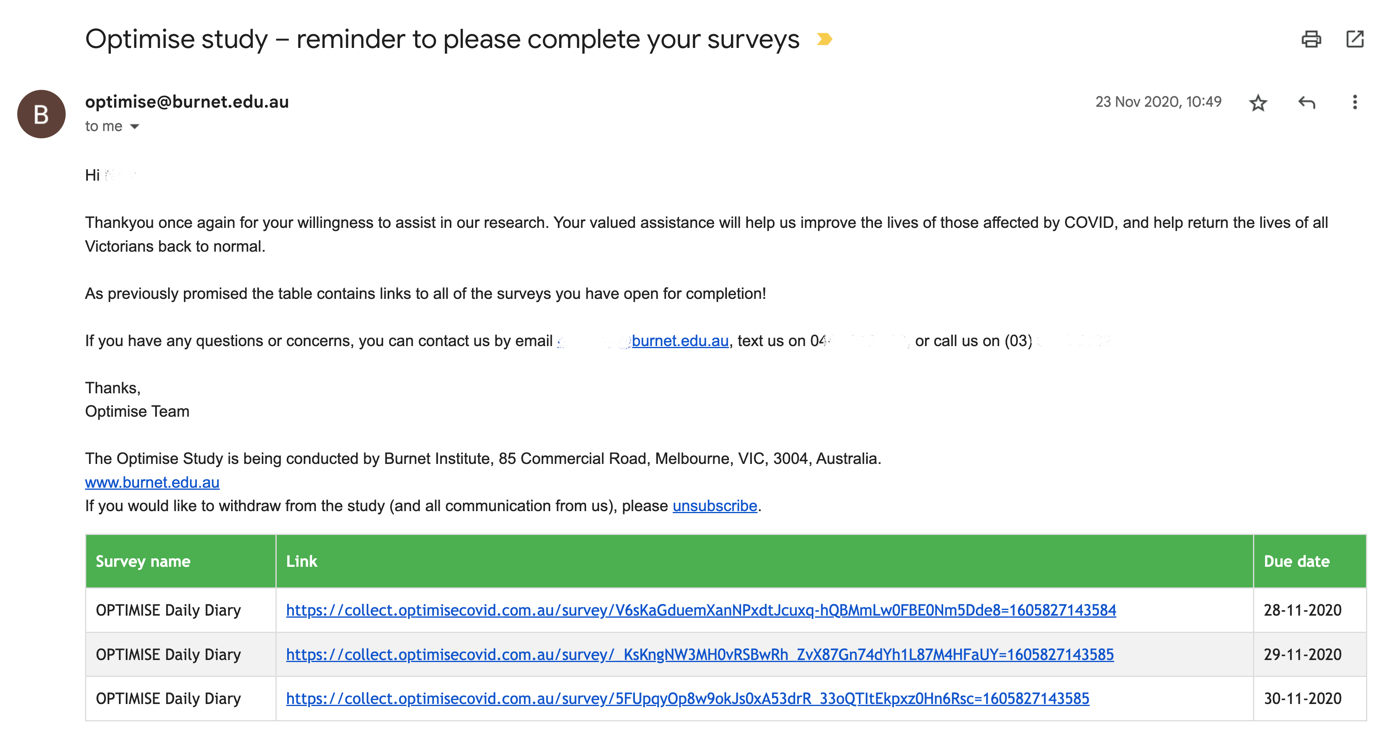
*
**Supplementary Figure *3*.** *An example of survey reminder email sent to a Optimise participant*.

Supplement: Supplementary file 3 — Additional file 3: Supplementary Figure 3. An example of survey reminder email sent to a Optimise participant. [file 12874_2023_1874_MOESM3_ESM.docx]

*
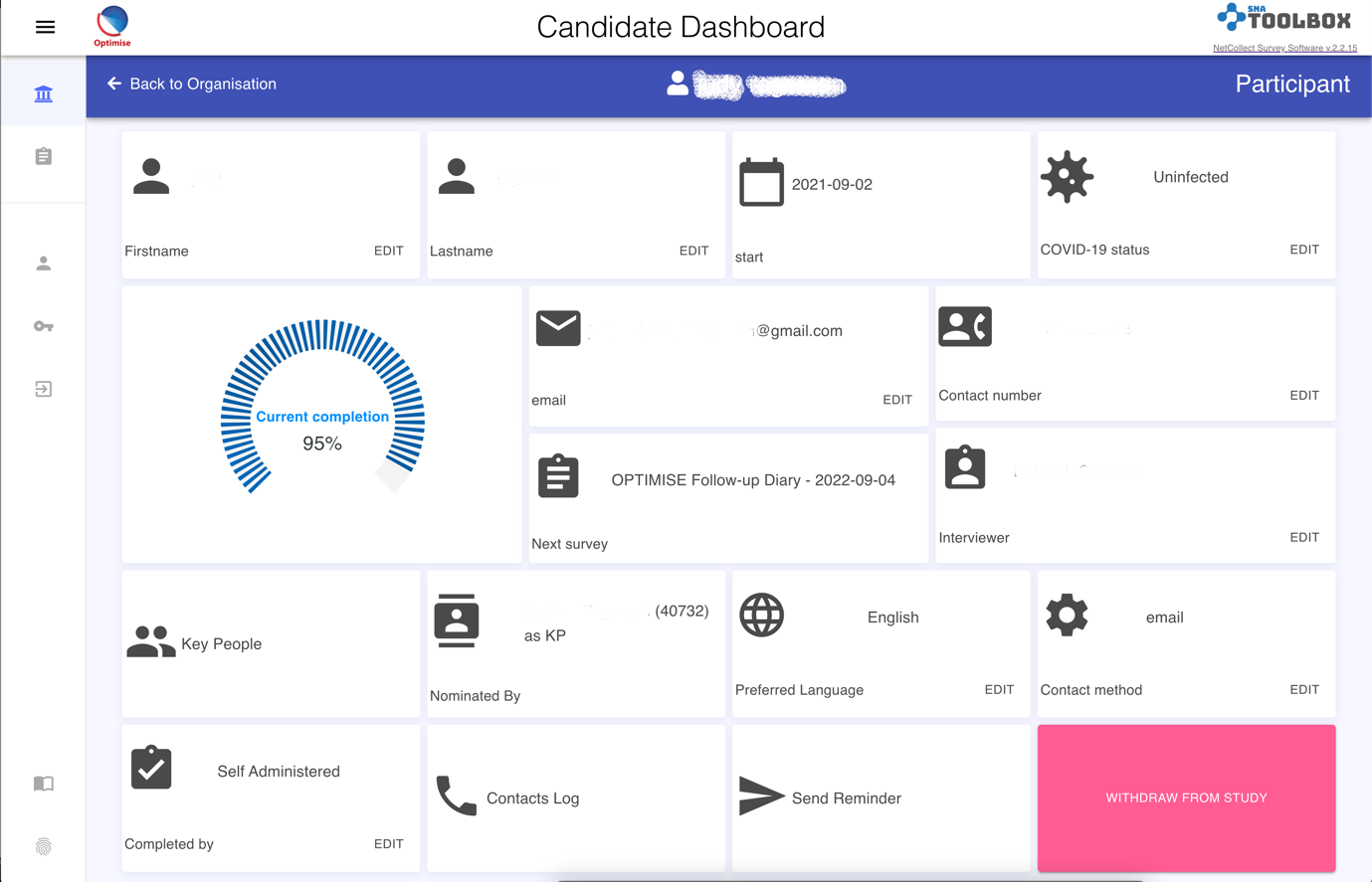
*

**Supplementary Figure 4.** An example of participant “dashboard” of a Optimise participant.

Supplement: Supplementary file 4 — Additional file 4: Supplementary Figure 4. An example of participant “dashboard” of a Optimise participant. [file 12874_2023_1874_MOESM4_ESM.docx]
