# Supplementary material for: Recruiting and retaining community-based participants in a COVID-19 longitudinal cohort and social networks study: lessons from Victoria, Australia
Source: BMC Med Res Methodol. 2023 Feb 27;23:54. doi: 10.1186/s12874-023-01874-z (PMC9969937; doi:10.1186/s12874-023-01874-z)
Supplement: Supplementary file 5 — Additional file 5: Supplementary Figure 5. An example of survey calendar shows survey completion of a Optimise participant. [file 12874_2023_1874_MOESM5_ESM.docx]

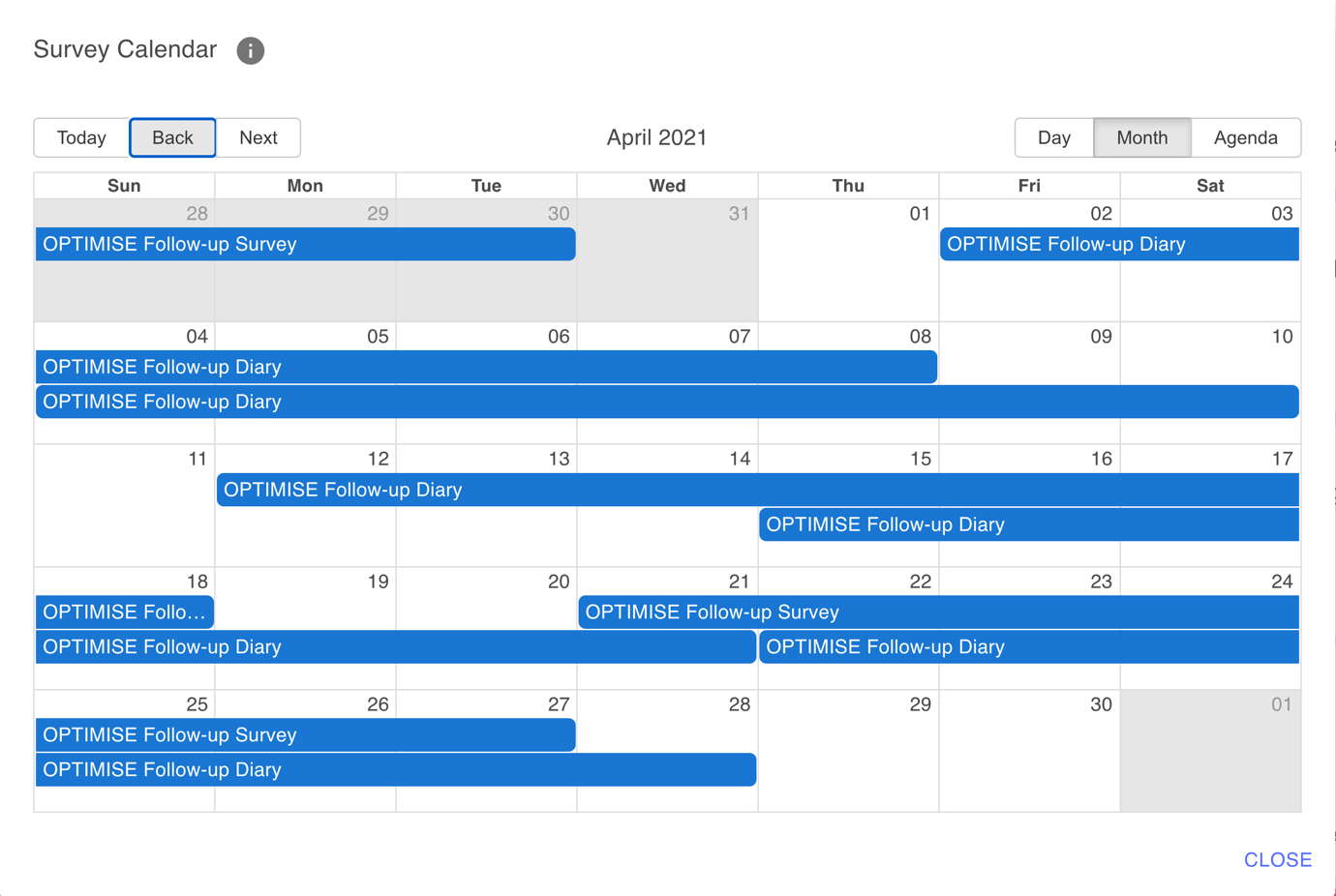

**Supplementary Figure 5.** An example of survey calendar shows survey completion of a Optimise participant.
